# Polymorphisms in *TLR4* Gene Associated With Somatic Cell Score in Water Buffaloes (*Bubalus bubalis*)

**DOI:** 10.3389/fvets.2020.568249

**Published:** 2020-11-05

**Authors:** Valentina Roldan-Montes, Diercles Francisco Cardoso, Naudin Alejandro Hurtado-Lugo, André Vieira do Nascimento, Daniel Jordan de Abreu Santos, Daiane Cristina Becker Scalez, Ana Cláudia de Freitas, Ana Cristina Herrera, Lucia Galvão Albuquerque, Gregório Miguel Ferreira de Camargo, Humberto Tonhati

**Affiliations:** ^1^Department of Animal Science, School of Agricultural and Veterinarian Science, São Paulo State University (UNESP), Jaboticabal, Brazil; ^2^Department of Animal Biosciences, Centre for Genetic Improvement of Livestock, University of Guelph, Guelph, ON, Canada; ^3^Facultad de Ciências Agrárias, Universidad Francisco de Paula Santander, Ocaña, Colombia; ^4^Department of Animal and Avian Sciences, University of Maryland, College Park, MD, United States; ^5^Escola de Medicina Veterinária e Zootecnia, Universidade Federal da Bahia, Salvador, Brazil

**Keywords:** milk yield, SNP, stop codon, udder health, molecular marker analysis, mastitis

## Abstract

Considering the importance of the diseases affecting the productive performance of animals in the dairy industry worldwide, it is necessary to implement tools that help to control and limit the occurrence of such diseases. As the increased somatic cell counts (SCC) are a direct expression of the inflammatory process, they are candidates to become the usual parameter for assessing udder health regarding milk quality and for monitoring mastitis incidences. Toll-Like Receptors are membrane proteins that play a key role in immunity, recognizing pathogens and, subsequently, activating immune responses. The present study was conducted to identify single nucleotide polymorphisms in the *TLR4* gene of buffaloes and to analyze its associations with somatic cell counts. DNA samples of 120 Murrah buffaloes were used. The whole coding region of the *TLR4* gene was amplified by polymerase chain reaction reactions and sequenced for polymorphism scanning. A total of 13 polymorphisms were identified for the sequenced regions of the *TLR4*, most of which are in the coding region. The association with the somatic cell score was highly significant (*p* < 0.001) for all identified polymorphisms of *TLR4* gene (g.54621T>A, g.54429G>T, g.54407T>A, g.46616C>A, g.46613T>G, g.46612A>G, g.46611C>A, g.46609T>G, g.46541C>G, g.46526C>A, g.46516T>C, g.46376C>T, g.46372T>C). Therefore, it is suggested that the markers of the *TLR4* gene can be used as molecular markers for mastitis resistance in buffaloes, due to their association with somatic cell counts.

## Introduction

The water buffalo (*Bubalus bubalis*) is a worldwide species used as a source of draft power, milk, and meat (1). The milk production represents an economically interesting activity, especially because of appreciated milk derivatives, such as “mozzarella,” and the remarkably yield efficiency manufacturing dairy products, due to buffalo's milk physical-chemical properties with high levels of protein, fat, and minerals (2–5). This species can be affected by health problems similar to those presented by cattle, among them the occurrence of mastitis (6), which consists of inflammatory reactions of the mammary gland triggered by the invasion of pathogens or traumatic events (7). The most frequently isolated pathogens in milk samples of buffaloes with mastitis are gram-positive bacteria, such as *Staphylococcus spp*. *Streptococcus spp*. (6, 8), and which causes mostly subclinical mastitis (9). However, low incidence of infection by Gram-negative bacteria, such as *Escherichia coli* and *Klebsiella pneumoniae*, has also been reported in the species (10, 11). Further than changes in milk composition, mastitis' major impacts on the dairy industry include the deterioration of animal comfort and welfare, the reduced performance of herds, increased therapeutic costs, and involuntary culling of cows early in lactation (12). Therefore, it is of paramount importance the development of strategies to reduce the incidence of this disease, such as the implementation of improved management practices and enhance of mastitis resistance in dairy herds through selection.

Subclinical mastitis, the most common inflammation of mammary tissue, is asymptomatic. Therefore, its diagnosis and onset of treatment rely largely on indirect indicators of inflammation, like the somatic cell count (SCC) test (13). The presence of somatic cells in milk is a normal physiological phenomenon even in healthy animals. Leukocytes and epithelial cells are constantly circulating in the mammary gland to guarantee its surveillance and protection against infection (14). However, increased SCC in the milk indicates the recruitment of the first line of cellular defense, particularly neutrophils, from blood to the mammary gland in response to long term intramammary inflammation caused by gram-positive bacteria (15). Analogously to bovine, the SCC limit of 200,000 cells/ml is usually adopted to define subclinical mastitis in buffaloes (6, 8, 14, 16), whereas values ranging between 11,000 and 171,000 cells/ml have been reported in the milk of healthy cows (11, 17). According to Salvador et al. (18) and Cerón-Muñoz et al. (17), the prevalence of subclinical mastitis in the Philippine and Brazilian water buffaloes herds was 42.8 and 3.2%, respectively.

Beyond being the most indicator of subclinical mastitis, SCC is widely applied as a selection criterion for enhanced resistance to mastitis, either subclinical or clinical (19). Although there is some controversy regarding the relation of low SCC and clinical mastitis resistance (13), studies in sheep and cattle have demonstrated that animals with higher SCC are most prone to mastitis (20, 21), and in agreement with these studies, the genetic correlation between SCC and clinical mastitis varying from moderate to high (0.59–0.77) have been reported in dairy cattle (22–24). In addition to its high correlation with clinical mastitis, the SCC represents an objective measure feasible of being periodically recorded in large populations with higher estimates of heritability than for clinical mastitis itself (25).

The comprehension of genomic regions and genes underlying the genetic variation in SCC of buffaloes might be useful for the assessment of genetic variability of mastitis resistance in the species, and it may assist the early selection of more robust animals. In addition, it is a pivotal first step for a better understanding of biological mechanisms behind the mastitis resistance and interpretation of the relation between clinical and subclinical mastitis, which are mostly associated with the activation of the innate and adaptive immune system, respectively (15). Toll-like receptor 4 (*TLR4*) is a pattern-recognition receptor that plays key roles stimulating the immune system by binding to pathogen-associated molecular patterns (PAMPs), such as lipopolysaccharide, an outer membrane component of gram-negative bacteria, as well as lipoteichoic acid present in the wall of some of the gram-positive bacteria (26, 27). Despite *TLR4* main importance to induce an innate immune response, it was an overexpressed gene in the mammary gland of bovine cows (28), and in the milk of buffaloes (29), infected exclusively by gram-positive bacteria. Therefore, *TLR4* corresponds to an interesting candidate gene for SCC, due its dual specificity to both gram-negative and –positive bacteria, hence its potential roles linking adaptive and innate immune systems (30). Moreover, Mesquita et al. (31) found a significant association between the polymorphisms of the *TLR4* and the SCC in Holstein cows, suggesting its potential to improve udder health through marker-assisted selection.

The present study aimed to verify the existence of polymorphisms in the coding region of the *TLR4* gene in a Brazilian buffalo population and verify its potential as molecular markers for infection resistance in the species, through assessing the association between *TLR4* polymorphisms and SCC in buffalo milk. In addition, we contrast the predicted protein structure of the *TLR4* gene of buffaloes and cattle.

## Materials and Methods

### Animals

The protocol for the present study was based on guidelines of the National Council for Animal Experimentation Control (CONCEA) and approved by the Committee on Ethics in the use of animals (CEUA) (protocol number 014624/17).

The phenotypic data and the biological material used in this study were provided by the Tapuio farm located in Taipu-RN, Brazil, which is part of the buffalo milk-recording program maintained by the Animal Science Department of the São Paulo State University (Unesp). The farms were free of brucellosis, tuberculosis, and leucosis and strictly follow the Brazilian vaccination calendar. The current herd has approximately 688 buffalo lactating dams, with average milk production of 2,042.11 ± 681.76 kg in up to 270 days of lactation. A total of 120 first-lactation cows that had milk sampled every other month during 2016 for determining the SCC were chosen for the genotyping process. The choice of the 120 cows considered a sampling that represented the genetic diversity of the herd and composed contemporary groups that contained at least three animals. The average of SCC in the milk analyses was 36,100 ± 586,000 cells/ml (ranging from 10,000 to 3,258,000 cells/ml). None of the animals presented clinical mastitis, only subclinical mastitis according to values of SCC exceeding 200,000 cells/ml. The large variation in the SCC makes the studied population a valuable source to initial evaluation of the association of the candidate gene *TLR4* and the udder-health indicator trait SCC.

Genomic DNA from 120 Murrah animals was extracted from tail hair follicles using the commercial extraction kit Macherey-Nagel NucleoSpin® Tissue (Düren, Germany).

### Primer Designs and PCR Reactions

The primer pairs used to amplify the coding region of the *TLR4* gene (Table 1) were designed using the Primer3 tool (http://bioinfo.ut.ee/primer3-0.4.0/), based on the *Bos taurus* sequence (GenBank accession number: AC_000165.1). The *TLR4* gene consists of 3 exons. Exons 1 and 3 include coding regions and the non-coding regions 5'UTR and 3'UTR, respectively. One pair of primers was designed for exons 1 and 2 each, whereas three further pairs of primers were designed for exon 3.

**Table 1:** Identifying the primers, the *TLR4* gene regions where the primers anneal, annealing temperature, and size of the amplified fragment.

**Primers**	Primers^*^	**T melting**	**Fragment**
**ID**		**(^**°**^C)**	**size (pb)**
1	F: 5′ ACAGGGAGAAGACAGCCAT 3′ R: 5′ CAAATGAACCTAACCAG 3′	54.4°C	728
2	F: 5′ AAATGAAGGGATAAGTG 3′ R: 5′ CTGAAGAAGGGAGAT 3′	43.3°C	535
3A^*^	F: 5′ TTTCATTTTGGTTTCCTAT 3′ R: 5′ TTATATCTTTGTTGTCTG 3′	49°C	894
3B^*^	F: 5′ CAAGGGTTGCTGTTCTCACA 3′ R: 5′ GGAAACTCTGATGTTC 3′	48.9°C	758
3C^*^	F: 5′ AAGGAGCAAGAACTACA 3′ R: 5′ AAAGAAGCACATCAGGGGA 3′	52.5°C	877

* *F, forward; R, reverse; 3A, 3B, 3C are the primers used in exon 3*.

The amplification of coding regions of the *TLR4* gene was performed by polymerase chain reaction (PCR) assays, which were conducted in Bio-rad S1000 thermal cyclers (Bio-Rad, Hercules, CA, USA) in a final volume of 15 μL, consisting of 100 ng of genomic DNA, 15 pM of each primer and the GoTaq Colorless MasterMix kit (Promega, Madison, WI, USA). The thermal profile was expressed by initial denaturation at 95°C for 5 min, followed by 34 cycles of denaturation at 95°C for 30 s; annealing at the melting temperature of each primer (Table 1) pair for 30 s; and extension at 72°C for 30 s. A final extension at 72°C for 5 min was performed. Further, 2 μL of PCR products were visualized by electrophoresis on 2% agarose gels stained with the GelRed system (Biotium, Inc., Hayward, CA, USA).

### Sequencing of Amplified Fragments

Each amplicon of the 120 samples was purified following the protocol recommended by the Wizard SV Gel and PCR Clean-Up System (Promega) kit. Then, they were sequenced from both primers (Forward and Reverse) by the dideoxynucleotide chain termination technique (ddNTPs) using the ABI PRISM BigDye Terminator Cycle Sequencing Ready Reaction Kit (Applied Biosystems) in an automatic ABI 3730 XL sequencer (Applied Biosystems). Then, the obtained DNA sequences were analyzed for identifying the polymorphisms using the CodonCode Aligner program. The structure of the *TLR4* buffalo protein was predicted by the Modeler software (32) while the three-dimensional structures of the proteins were generated by the UCSF Chimera (33).

### Allele and Genotype Frequencies

The allele and genotype frequencies of the polymorphisms detected in the *TLR4* gene were estimated for the population by counting. The Hardy-Weinberg equilibrium was verified for each polymorphic site by the chi-square test (χ^2^) (*p* < 0.05). The linkage disequilibrium between the pairs of markers was estimated from the *r*^2^ statistics (34) using the Plink software (35).

### Association Analysis

The SCC evaluated in the association study refers to the first lactation records of 120 dairy buffaloes. The association analyses were performed by a mixed model using univariate analyzes in the Proc Mixed procedure of the SAS/STAT 9.3 statistical program (SAS Institute, Inc., Cary, NC, USA). The following statistical model was applied:

$$\begin{array}{l} y_{ijkl}=\mu +CG_{i}+\;b_{1}IV_{j}+b_{2}IV_{j}^{2}+M_{l}+T_{k}+e_{ijkl} \end{array}$$

where, *y*_*ijkl*_ is the SCC determined for the ijkl^th^ animal; μ is the average of the SCC in the population; *CG*_i_ is the fixed effect of i^th^ contemporary group (year and season of birth); *IV*_*j*_ is the age of the j^th^ dam at birth, considered as a linear and quadratic (co)variable in the model; *M*_*l*_is the fixed effect of the l^th^ genotype for each polymorphism detected in the *TLR4* gene; *T*_*k*_ is the random effect of the sire, and *e*_*ijkl*_ is the residual random effect associated with the observation *y*_*ijkl*_.

Since the SCC data do not have a normal distribution, the generalized linear mixed model was performed assuming Poisson distribution for the trait. To evaluate the association of the marker with the studied trait, a significance threshold for the *P*-value was calculated by Bonferroni correction (α = 0.05/N_polymorphisms_). The additive and the dominance effects of the significant SNPs were tested after analyzing the associations between the SNPs and SCC. These analyses were performed by orthogonal contrasts using the PROC GLM in SAS (SAS 9.2, SAS Institute, Cary, NC, USA).

The probabilities of haplotypes were constructed in the Haploview software to analyze and visualize linkage disequilibrium patterns (36), using the block system reported by Gabriel et al. (37) where the region is segmented according to the LD.

## Results and Discussion

A total of 13 SNPs were identified in the studied population (Table 2), three in exon 1 and 10 in exon 3 of the *TLR4* gene. The SNP g.54621T>A of exon 1 was the only one located in a non-coding region (5'UTR), whereas the SNP g.46616C>A|T, of the same exon, was the only polymorphism with more than two alternative alleles. The three alleles of this *locus* lead to three different amino acids in the coded protein, increasing the protein variability level. Moreover, some of the identified polymorphisms were located in the same codon (e.g., g.46611C>A, g.46612A>G and g.46613T>G), which increases the number of possible nucleotide combination in a codon and also contributes to the protein variability. Almost all SNPs found in the *TLR4* gene have non-synonymous conservative amino acid substitutions in the protein. The fact that the *TLR4* gene is a cell-surface pattern-recognition receptor important to recognition of a broad of PAMPs, somehow justifies the amount of SNPs and the variability of predicted protein isoforms detected in this sample. White et al. (38) also reported a relative abundance of polymorphism (*N* = 28) in the coding region *TLR4* gene in a sample involving 11 different cattle breeds. Similarly, a large amount of polymorphisms has been shown in the coding region of *TLR4* in human populations (*N* > 50) (39). Consulting the Ensembl variation resource (40), we observed that 224, 61, and 36 variations have been described in the coding region of *TLR4* in cattle (ENSBTAT00000008190.3), sheep (ENSOART00000006304.1), and goat (ENSCHIT00000027327.1), respectively.

**Table 2:** SNPs and amino acid changes in the *TLR4* gene.

**Exon**	SNP^*^	**Amino acid change**
1	g.54621T>A	–
1	g.54429G>T	Thr/Met
1	g.54407T>A	Arg/Ser
3	g.46616C>A	Cys/Tyr
3	g.46613T>G	Tyr/Cys
3	g.46612A>G	Tyr/Stp
3	g.46611C>A	Glu/Lys
3	g.46609T>G	Glu/Glu
3	g.46541C>G	Gly/Asp
3	g.46526C>A	Gly/Asp
3	g.46516T>C	Lys/Asn
3	g.46376C>T	Arg/Ile
3	g.46372T>C	Leu/Phe

* *Sequence-based marker positioning >NW_005784801.1*.

A noteworthy variation in the population refers to the possible combination of the alleles A and T of the SNPs g.46612A>G and g.46613T>G, respectively. This allelic combination corresponds to the establishment of a premature stop codon, which causes the interruption of the protein synthesis with 191 amino acids instead of the 841 expected under normal synthesis (41, 42). Surprisingly, 3 of the healthy cows presented two copies of haplotype AT (i.e., AA in SNP g.46612A>G and TT in the g.46613T>G), which corresponds to two copies of the premature stop codon and absence of functional *TLR4* protein. Despite the direct influence of *TLR4* in the immune system, this non-sense mutation did not represent a recessive lethal combination, although the truncated protein did not completely encompass the predicted signaling domain of its wild type *TLR4* (43). It is questionable that these animals have survived up to adult age without being naturally exposed to gram-negative bacterial infections. Therefore, our findings raise a point to be further investigated about the biological mechanisms compensating the *TLR4* absence in these buffaloes samples when it becomes essential. It is noteworthy that the two adjacent SNPs g.46612A>G and g.46613T>G presented the lowest linkage disequilibrium measured by *r*^2^ between pairwise of SNPs in exon 3 (*r*^2^ = 0.07, Table 3), which might represent purifying selection frequently acting on the stop codon determinant haplotypes, AT.

**Table 3:** Estimates of linkage disequilibrium (*r*^2^) of the *TLR4* SNPs.

**SNP**	**g.54429** **G>T**	**g.54407** **T>A**	**g.46616** **C>A**	**g.46613** **T>G**	**g.46612** **A>G**	**g.46611** **C>A**	**g.46609** **T>G**	**g.46541** **C>G**	**g.46526** **C>A**	**g.46516** **T>C**	**g.46376** **C>T**	**g.46372** **T>C**
g.54621T>A	0.929	0.946	0.008	0.011	0.002	0.012	0.255	0.014	0.023	0.032	0.003	0.008
g.54429G>T		0.982	0.017	0.022	0.004	0.016	0.030	0.021	0.031	0.026	0.010	0.010
g.54407T>A			0.017	0.023	0.004	0.019	0.028	0.020	0.030	0.027	0.009	0.009
g.46616C>A				0.155	0.726	0.737	0.648	0.800	0.852	0.246	0.774	0.678
g.46613T>G					0.071	0.171	0.197	0.165	0.144	0.089	0.142	0.176
g.46612A>G						0.594	0.588	0.763	0.758	0.311	0.671	0.713
g.46611C>A							0.789	0.748	0.764	0.243	0.706	0.651
g.46609T>G								0.759	0.770	0.277	0.648	0.708
g.46541C>G									0.916	0.305	0.828	0.849
g.46526C>A										0.302	0.806	0.828
g.46516T>C											0.251	0.322
g.46376C>T												0.796

**Figure 1:**
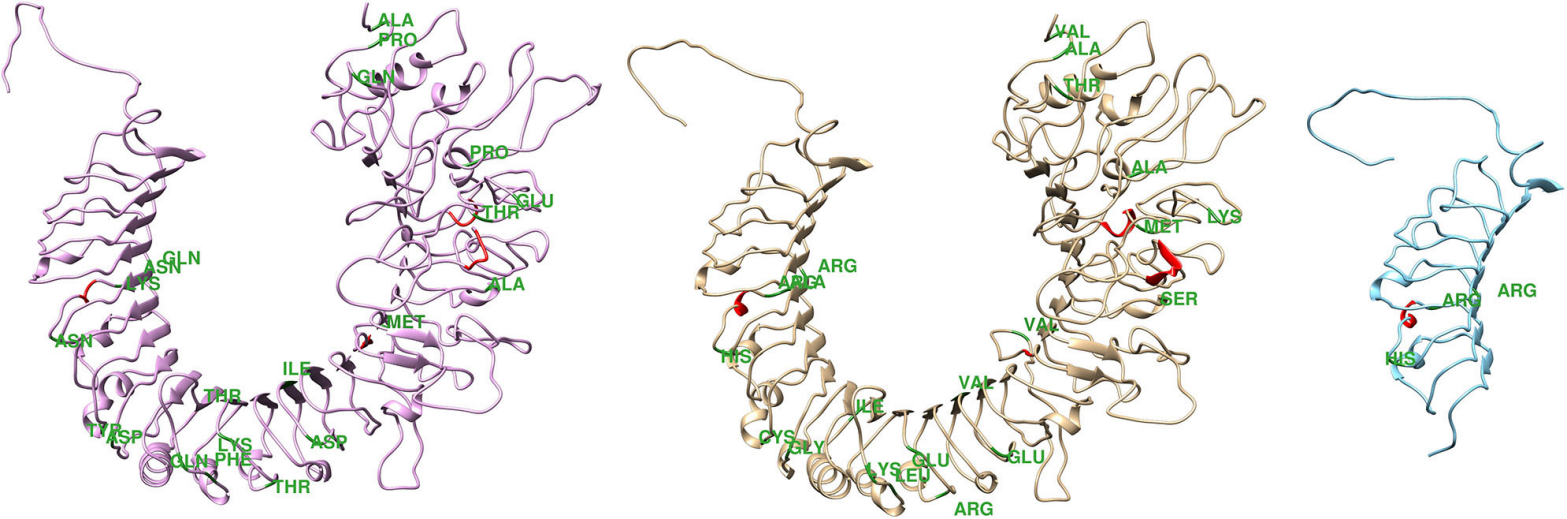
Theoretical three-dimensional model for bovine and buffalo proteins. From right to left are represented the predicted structure of the bovine protein, buffalo protein in the absence and presence of premature stop codon. * Green indicates amino acid exchanges.

Beyond the SNPs in the buffalo species, the sequencing of the amplicons revealed 45 punctual differences in coding regions across buffaloes and cattle (Supplementary Table 1), 20 of them resulting in amino acids alterations. Some of the across species missense variation were non-conservative, suggesting that structural and functional differences exist between the species (44). Figure 1 shows the three-dimensional protein structure predicted for each bovine and buffalo species, as well as the predicted structure in the presence of a premature stop codon. It has been postulated that buffaloes are less susceptible to bacterial infection than cattle, due to some anatomic attributes, such as their longer narrow teat compared to bovine, which prevents pathogens invasion (6). The structural difference in proteins encoded by *TLR4* genes of the two species might represent an additional physiological mechanism empowering this difference in mastitis resistance.

The allele and genotype frequencies of each SNP are shown in Table 4. Most SNPs had minor allele frequency (MAF) higher than 0.10, which is favorable to performing association analyses since low MAF could affect the prediction of SNP effects (45). Most of the SNP frequencies adhered to the expected value of the Hardy-Weinberg Equilibrium verified by the chi-square test (χ^2^) (*P* < 0.05). However, two of them were not in equilibrium, including the SNP g.46612A>G that is related to the premature stop codon. Another indication that purifying selection, or other of the factors that affect Hardy-Weinberg Equilibrium, might be acting over this particular *locus*.

**Table 4:** Allele and genotype frequencies and Hardy-Weinberg equilibrium of the polymorphisms found in the *TLR4* gene.

**SNP**	**Allele frequency**		**Genotype frequency**			**H-W equilibrium**
	**T**	**A**	**TT**	**TA**	**AA**	***χ***^2^
g.54621T>A	0.2864	0.7146	0.0971	0.3786	0.5243	0.4715
	**G**	**T**	**TT**	**GT**	**GG**	
g.54429G>T	0.7048	0.2952	0.5192	0.3846	0.1058	0.3585
	**A**	**T**	**AA**	**AT**	**TT**	
g.54407T>A	0.7165	0.2835	0.1134	0.3402	0.5464	0.1316
	**A**	**C**	**AA**	**AC**	**CC**	
g.46616C>A	0.5818	0.4182	0.3727	0.4182	0.2091	0.1692
	**T**	**G**	**TT**	**TG**	**GG**	
g.46613T>G	0.9204	0.0797	0.8496	0.1416	0.0088	0.5203
	**A**	**G**	**AA**	**AG**	**GG**	
g.46612A>G	0.4789	0.5211	0.3474	0.2632	0.3895	4.067^*^
	**C**	**A**	**CC**	**CA**	**AA**	
g.46611C>A	0.65	0.35	0.50	0.30	0.20	0.0006
	**T**	**G**	**TT**	**TG**	**GG**	
g.46609T>G	0.3785	0.6215	0.4299	0.3832	0.1869	0.0639
	**C**	**G**	**CC**	**CG**	**GG**	
g.46541C>G	0.5841	0.4159	0.3540	0.4602	0.1858	0.5656
	**C**	**A**	**CC**	**AC**	**AA**	
g.46526C>A	0.4306	0.5694	0.2222	0.4167	0.3611	0.1195
	**T**	**C**	**TT**	**TC**	**CC**	
g.46516T>C	0.6712	0.3288	0.26	0.37	0.37	3.382
	**C**	**T**	**CC**	**CT**	**TT**	
g.46376C>T	0.5585	0.4415	0.3723	0.3723	0.2553	0.0209
	**T**	**C**	**TT**	**CT**	**CC**	
g.46372T>C	0.4307	0.5693	0.2277	0.4059	0.3663	0.1034

* *SNP not in Hardy-Weinberg equilibrium*.

**Figure 2:**
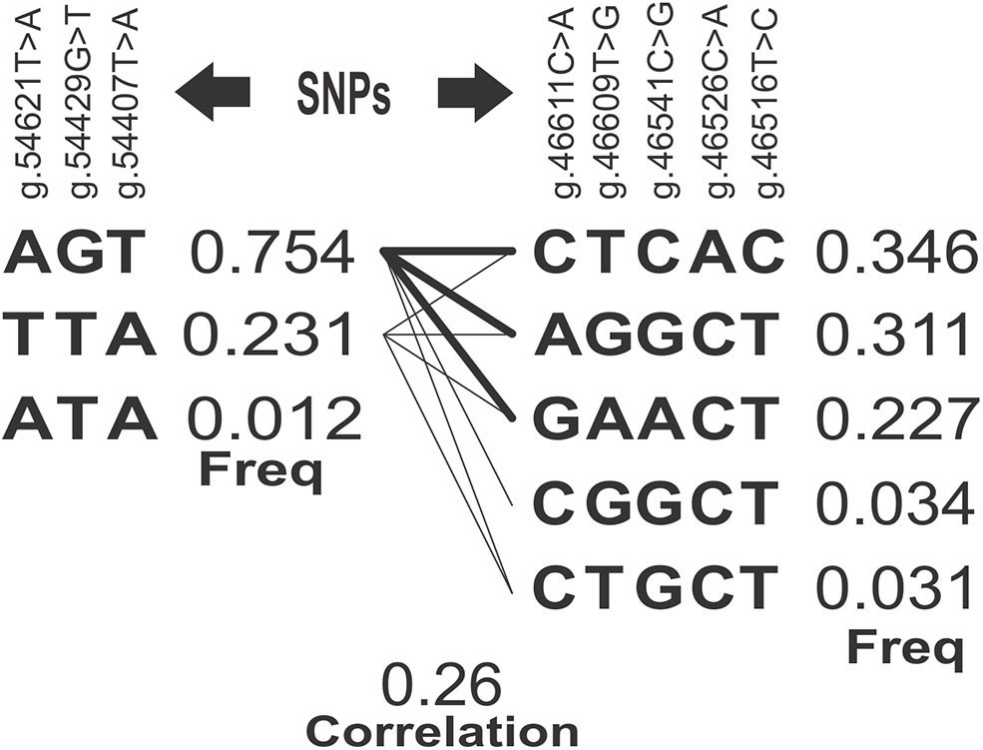
Blocks of haplotypes estimated correlations and frequencies. SNPs, Single Nucleotide Polymorphism; Freq, Frequency estimated for each haplotype.

SNPs with higher *r*^2^ tend to segregate together because they are closer. The LD of the g.54621T>A, g.54429G>T, and g.54407T>A SNPs (Table 3) suggested very high concordance between the genotypes of three *loci* (0.929, 0.946, 0.982), indicating that their alleles segregate together. Therefore, any of the three SNPs might equally capture the proportion of the genetic variation of the SCC trait, which is attributed to a causative mutation close/among them or to the block. Accordingly, five SNPs of the exon 3 (g.46611C>A, g.46609T>G, g.46541C>G, g.46526C>A, and g.46516T>C) also represented a block in high LD. The correlation between the blocks (0.26) and the estimated frequencies for each of the haplotypes is shown in Figure 2, being ACA and GAAAC the most common haplotypes in exons 1 and 3 with estimated frequencies of 0.75 and 0.34, respectively.

Adjusted means of SCC were compared according to the genotype classes of each SNP, and significant differences (*P* < 0.0001) were observed in all *loci* (Table 5). A significant effect of allele substitution was observed for all SNPs, indicating phenotypical differences associated with the number of copies of favorable alleles in each SNP, even if some of them are not the causative mutations. As selection for SCC would target the lower values, due to its association with great mastitis resistance, the alleles related to the reduction in the averages would be favorable, e.g., the allele T of SNP g.54621T>A and the allele A of the SNP g.46616C>A. Roughly, for SNP g.54621T>A, where the allele substitution was modeled as replace of alleles Ts by As (“T>A” representing TT > TA >AA), the SNP effect represents the regression coefficient of SCC on the genotypes TT, TA, AA, hence the substitution effect of A allele (Table 5). Additionally, some SNPs showed a significant dominance effect, including g.46616C>A, g.46611C>A, g.46541C>G, and g.46526C>A.

**Table 5:** Adjusted means and standard errors of somatic cell counts (SCC, 1,000/ml) per genotypes, substitution allele, and dominance effect.

**SNP**	Averages of genotypes^*^			**Allele effect**	***P***	**Dominance effect**	***P***
	**TT**	**AT**	**AA**				
g.54621T>A	4.81 ± 0.34^b^	5.65 ± 0.34^a^	5.56 ± 0.34^a^	0.44 ± 0.01	<0.0001	0.01 ± 0.13	0.98
	**GG**	**GT**	**TT**				
g.54429G>T	4.86 ± 0.31^c^	5.47 ± 0.31^b^	5.63 ± 0.31ª	0.42 ± 0.01	<0.0001	0.02 ± 0.13	0.86
	**TT**	**AT**	**AA**				
g.54407T>A	5.73 ± 0.37^a^	5.74 ± 0.37ª	4.76 ± 0.37^b^	0.51 ± 0.01	<0.0001	−0.03 ± 0.14	0.83
	**CC**	**AC**	**AA**				
g.46616C>A	5.73 ± 0.30^a^	5.48 ± 0.30^b^	4.86 ± 0.30^c^	−0.41 ± 0.01	<0.0001	−0.28 ± 0.01	<0.0001
	**TT**	**TG**	**GG**				
g.46613T>G	5.27 ± 0.26^b^	5.80 ± 0.26^a^	6.13 ± 0.99^a^	0.56 ± 0.02	<0.0001	0.04 ± 0.17	0.82
	**AA**	**AG**	**GG**				
g.46612A>G	5.92 ± 0.33ª	4.61 ± 0.33^b^	4.33 ± 0.33^c^	−0.53 ± 0.01	<0.0001	**–**0.12 ± 0.15	0.43
	**CC**	**CA**	**AA**				
g.46611C>A	5.00 ± 0.28ª	5.38 ± 0.28^b^	5.70 ± 0.28^c^	0.29 ± 0.01	<0.0001	0.08 ± 0.01	<0.0001
	**TT**	**TG**	**GG**				
g.46609T>G	4.88 ± 0.29^c^	5.43 ± 0.29^b^	6.18 ± 0.29^a^	0.49 ± 0.01	<0.0001	0.05 ± 0.12	0.66
	**CC**	**CG**	**GG**				
g.46541C>G	4.76 ± 0.31^c^	5.46 ± 0.31^b^	5.86 ± 0.31^a^	0.47 ± 0.01	<0.0001	0.20 ± 0.01	<0.0001
	**CC**	**CA**	**AA**				
g.46526C>A	6.06 ± 0.36ª	5.37 ± 0.36^b^	4.96 ± 0.36^c^	−0.24 ± 0.01	<0.0001	−0.07 ± 0.01	<0.0001
	**TT**	**AT**	**CC**				
g.46516T>C	5.79 ± 0.25ª	4.68 ± 0.25^b^	4.59 ± 0.25^c^	−0.57 ± 0.01	<0.0001	−0.11 ± 0.16	0.48
	**CC**	**CT**	**TT**				
g.46376C>T	5.03 ± 0.2^b^	4.84 ± 0.2^c^	5.53 ± 0.2^a^	−0.03 ± 0.01	<0.0001	−0.12 ± 0.13	0.38
	**TT**	**TC**	**CC**				
g.46372T>C	4.72 ± 0.28^c^	5.21 ± 0.28^b^	5.85 ± 0.28ª	0.21 ± 0.01	<0.0001	0.07 ± 0.13	0.58

* *a, b, c different letters in the same row indicate significant differences (p <0.006) by the Bonferroni test. p, p-value*.

To the best of our knowledge, it is the first study of the effect of SNP in the *TRL4* gene on the SCC in buffaloes, demonstrating that *TRL4* polymorphisms are associated with the SCC concentration in the milk of healthy animals. Only a few studies regarding candidate genes for economically important traits are available in buffaloes (46–49). For mastitis resistance, they are even scarcer (50). Alfano et al. (51) reported significant results for the tuberculosis resistance of SNPs in the *TRL4* gene of dairy buffaloes. The SNP (672A>C) described by these authors is the same reported here, as SNP g.46516T>C. It evidences the importance of gene polymorphisms in the immune system of ruminants. In cattle, *TLR4* polymorphisms have been associated with SCC (31, 52). Thus, despite the large number of non-synonymous divergences between the amino acids in the *TLR4* of buffaloes and bovine, the roles of the gene in the immune response of the mammary gland is preserved in both species.

DNA polymorphism can be used to assist the selection for a specific trait when it is associated with such traits. Specific markers can also be used to avoid some congenital recessive defects of genetic origin that affects cattle, such as bovine leukocyte adhesion deficiency (BLAD, 40). Considering the impact of mastitis in milk production, the usefulness of SCC as an indicator of mastitis resistance, and the results found in this study, there is evidence that the *TLR4* gene can be used for marker-assisted selection. We need to emphasize that even finding promising results, this is a preliminary study with a small population. Further studies involving a larger sample, more generations, and animals with clinical mastitis are encouraged to validate these results. Additionally, the milk cell profile of cows that were homozygous for the premature stop codon, as well as the animal reaction face bacterial challenge are yet to be adequately assessed. The intriguing point behind the stop codon detected in the current study is the well-known importance of *TLR4* for the immune system, especially for immune responses to gram-negative bacteria. Other non-lethal premature stop codons have been reported for other genes in the literature. For instance, a non-sense mutation was also reported in buffaloes *JY-1*, which encodes an oocyte-specific protein. Additionally, a premature stop codon created a novel favorable allele for muscle development in the *GDF8* of cattle (53).

The *TLR4* buffalo gene shows a different coding region in comparison to the cattle sequence with many non-synonymous polymorphisms. *TLR4* gene is highly polymorphic in buffaloes, especially for exon 3. The non-synonymous polymorphisms herein presented show potential as molecular markers for SCC in buffaloes and likely for resistance to clinical mastitis.

## Data Availability Statement

The datasets presented in this study can be found in online repositories. The names of the repository/repositories and accession number(s) can be found at: https://www.ncbi.nlm.nih.gov/, NW_005784801.1.

## Ethics Statement

The animal study was reviewed and approved by National Council for Animal Experimentation Control (CONCEA). Written informed consent was obtained from the owners for the participation of their animals in this study.

## Author Contributions

VR-M, NH-L, GdC, and HT planned the study. VR-M, DC, AdN, and AdF performed the molecular. NH-L, AH, DS, and DSa performed the curation of data and statistical analyses. LA and DC supported the data analysis and contributed to the interpretation of the results. VR-M composed the original draft. All authors read and approved the current draft.

## Conflict of Interest

The authors declare that the research was conducted in the absence of any commercial or financial relationships that could be construed as a potential conflict of interest.

## References

[1] Bernardes O Buffaloes breeding in Brasil. Ital J Anim Sci. (2007) 6:162–7. 10.4081/ijas.2007.s2.162

[2] Valle JLE Características e usos do leite de bubalinos. Reun Anu da Soc Bras Zootec. (1990) 27:739–43.

[3] Dubey PC Suman CL Sanyal MK Pandey HS Saxena MM Yadav PL Factors affecting composition of milk of buffaloes. Indian J Anim Sci. (1997) 67:802–4.

[4] Tonhati H Albuquerque LG de Oliveira JFS Melhoramento genético em bubalinos. Programa Vale do Ribeira, SP. Simpósio Nac Melhor Genético Anim. (1996) 1:69–72.

[5] Patel RS Mistry VV Physicochemical and structural properties of ultrafiltered buffalo milk and milk powder1. J Dairy Sci. (1997) 80:812–7. 10.3168/jds.S0022-0302(97)76002-8

[6] Moroni P Rossi CS Pisoni G Bronzo V Castiglioni B Boettcher PJ. Relationships between somatic cell count and intramammary infection in buffaloes. J Dairy Sci. (2006) 89:998–1003. 10.3168/jds.S0022-0302(06)72165-8 16507694

[7] Bedolla CC de León MERP Pérdidas económicas ocasionadas por la mastitis bovina en la industria lechera. Rev Electrónic Vet. (2008) 9:1–26.

[8] Dhakal IP. Normal somatic cell count and subclinical mastitis in murrah buffaloes. J Vet Med Ser B. (2006) 53:81–6. 10.1111/j.1439-0450.2006.00918.x 16626405

[9] Saglam AG Sahin M Çelik E Çelebi Ö Akça D Otlu S. The role of staphylococci in subclinical mastitis of cows and lytic phage isolation against to *Staphylococcus aureus*. Vet World. (2017) 10:1481–5. 10.14202/vetworld.2017.1481-1485 29391690 PMC5771174

[10] Preethirani PL Isloor S Sundareshan S Nuthanalakshmi V Deepthikiran K Sinha AY. Isolation, biochemical and molecular identification, and *in-vitro* antimicrobial resistance patterns of bacteria isolated from bubaline subclinical mastitis in South India. PLoS ONE. (2015) 10:e0142717. 10.1371/journal.pone.0142717 26588070 PMC4654528

[11] Vásquez-García A Silva TDS de Almeida-Queiroz SR Godoy SHS Fernandes AM Sousa RLM Species identification and antimicrobial susceptibility profile of bacteria causing subclinical mastitis in buffalo. Pesqui Vet Bras. (2017) 37:447–52. 10.1590/s0100-736x2017000500004

[12] Heringstad B Egger-Danner C Charfeddine N Pryce JE Stock KF Kofler J. Invited review: genetics and claw health: opportunities to enhance claw health by genetic selection. J Dairy Sci. (2018) 101:4801–21. 10.3168/jds.2017-13531 29525301

[13] Rainard P Foucras G Boichard D Rupp R. Invited review: low milk somatic cell count and susceptibility to mastitis. J Dairy Sci. (2018) 101:6703–14. 10.3168/jds.2018-14593 29803421

[14] Alhussien MN Dang AK. Milk somatic cells, factors influencing their release, future prospects, and practical utility in dairy animals: an overview. Vet World. (2018) 11:562–77. 10.14202/vetworld.2018.562-577 29915493 PMC5993762

[15] Heringstad B Klemetsdal G Ruane J Selection for mastitis resistance in dairy cattle: a review with focus on the situation in the nordic countries. Livest Prod Sci. (2000) 64:95–106. 10.1016/S0301-6226(99)00128-1

[16] Schukken YH Wilson DJ Welcome F Garrison-Tikofsky L Gonzalez RN. Monitoring udder health and milk quality using somatic cell counts. Vet Res. (2003) 34:579–96. 10.1051/vetres:2003028 14556696

[17] Cerón-Muñoz M Tonhati H Duarte J Oliveira J Muñoz-Berrocal M Jurado-Gámez H. Factors affecting somatic cell counts and their relations with milk and milk constituent yield in buffaloes. J Dairy Sci. (2002) 85:2885–9. 10.3168/jds.S0022-0302(02)74376-2 12487456

[18] Salvador R Beltran J Abes N Gutierrez C Mingala C. Short communication: prevalence and risk factors of subclinical mastitis as determined by the California mastitis test in water buffaloes (*Bubalis bubalis*) in Nueva Ecija, Philippines. J Diary Sci. (2012) 95:1363–6. 10.3168/jds.2011-4503 22365218

[19] Rupp R Beaudeau F Boichard D. Relationship between milk somatic-cell counts in the first lactation and clinical mastitis occurrence in the second lactation of French holstein cows. Prev Vet Med. (2000) 46:99–111. 10.1016/S0167-5877(00)00142-2 10878298

[20] Koeck A Miglior F Kelton DF Schenkel FS. Alternative somatic cell count traits to improve mastitis resistance in Canadian holsteins. J Dairy Sci. (2012) 95:432–9. 10.3168/jds.2011-4731 22192222

[21] Rupp R Bergonier D Dion S Hygonenq MC Aurel MR Robert-Granié C. Response to somatic cell count-based selection for mastitis resistance in a divergent selection experiment in sheep. J Dairy Sci. (2009) 92:1203–19. 10.3168/jds.2008-1435 19233814

[22] Koivula M Mä EA Negussie E Serenius T. Genetic and phenotypic relationships among milk yield and somatic cell count before and after clinical mastitis. J Dairy Sci.(2005) 88:827–33. 10.3168/jds.S0022-0302(05)72747-8 15653550

[23] Koeck A Heringstad B Egger-Danner C Fuerst C Winter P Fuerst-Waltl B. Genetic analysis of clinical mastitis and somatic cell count traits in Austrian Fleckvieh cows. J Dairy Sci. (2010) 93:5987–95. 10.3168/jds.2010-3451 21094773

[24] Govignon-Gion A Dassonneville R Baloche G Ducrocq V. Multiple trait genetic evaluation of clinical mastitis in three dairy cattle breeds. Animal. (2016) 10:558–65. 10.1017/S1751731115002529 26592099

[25] Martin P Barkema HW Brito LF Narayana SG Miglior F. Symposium review: Novel strategies to genetically improve mastitis resistance in dairy cattle. J Dairy Sci. (2018) 101:2724–36. 10.3168/jds.2017-13554 29331471

[26] Takeuchi O Hoshino K Kawai T Sanjo H Takada H Ogawa T. Differential roles of TLR2 and TLR4 in recognition of gram-negative and gram-positive bacterial cell wall components. Immunity. (1999) 11:443–51. 10.1016/S1074-7613(00)80119-3 10549626

[27] Branger J Knapp S Weijer S Leemans JC Pater JM Speelman P. Role of toll-like receptor 4 in gram-positive and gram-negative pneumonia in mice. Infect Immun. (2004) 72:788–94. 10.1128/IAI.72.2.788-794.2004 14742522 PMC321591

[28] Goldammer T Zerbe H Molenaar A Schuberth HJ Brunner RM Kata SR Mastitis increases mammary mRNA abundance of β-defensin 5, toll-like-receptor 2 (TLR2), and TLR4 but not TLR9 in cattle. Clin Diagn Lab Immunol. (2004) 11:174–85. 10.1128/CDLI.11.1.174-185.2004 14715566 PMC321333

[29] Tanamati F Stafuzza NB Gimenez DFJ Stella AAS Santos DJA Ferro MIT. Differential expression of immune response genes associated with subclinical mastitis in dairy buffaloes. Animal. (2019) 13:1651–7. 10.1017/S1751731118003324 30621802

[30] Raffeiner B Dejaco C Duftner C Kullich W Goldberger C Vega SC. Between adaptive and innate immunity: TLR4-mediated perforin production by CD28null T-helper cells in ankylosing spondylitis. Arthritis Res Ther. (2005) 7:R1412. 10.1186/ar1840 16277694 PMC1297589

[31] Mesquita AQ de Mesquita AJ de Jardim EAG da V Kipnis APJ. Association of TLR4 polymorphisms with subclinical mastitis in Brazilian holsteins. Brazilian J Microbiol. (2012) 43:692–7. 10.1590/S1517-83822012000200034 24031881 PMC3768839

[32] Šali A Blundell TL. Comparative protein modelling by satisfaction of spatial restraints. J Mol Biol. (1993) 234:779–815. 10.1006/jmbi.1993.1626 8254673

[33] Pettersen EF Goddard TD Huang CC Couch GS Greenblatt DM Meng EC. UCSF Chimera—a visualization system for exploratory research and analysis. J Comput Chem. (2004) 25:1605–12. 10.1002/jcc.20084 15264254

[34] Hill WG Robertson A. The effect of linkage on limits to artificial selection. Genet Res. (1966) 8:269–94. 10.1017/S0016672300010156 5980116

[35] Purcell S Neale B Todd-Brown K Thomas L Ferreira MA Bender D. PLINK: a tool set for whole-genome association and population-based linkage analyses. Am J Hum Genet. (2007) 81:559–75. 10.1086/519795 17701901 PMC1950838

[36] Barrett JC Fry B Maller J Daly MJ. Haploview: analysis and visualization of LD and haplotype maps. Bioinformatics. (2004) 21:263–5. 10.1093/bioinformatics/bth457 15297300

[37] Gabriel SB Schaffner SF Nguyen H Moore JM Roy J Blumenstiel B. The structure of haplotype blocks in the human genome. Science. (2002) 296:2225–9. 10.1126/science.1069424 12029063

[38] White SN Taylor KH Abbey CA Gill CA Womack JE. Haplotype variation in bovine Toll-like receptor 4 and computational prediction of a positively selected ligand-binding domain. Proc Natl Acad Sci USA. (2003) 100:10364–9. 10.1073/pnas.1333957100 12915733 PMC193567

[39] Smirnova I Hamblin MT McBride C Beutler B Di Rienzo A. Excess of rare amino acid polymorphisms in the toll-like receptor 4 in humans. Genetics. (2001) 158:1657–64. 11514453 10.1093/genetics/158.4.1657 PMC1461767

[40] Hunt SE McLaren W Gil L Thormann A Schuilenburg H Sheppard D. Ensembl variation resources. Database. (2018) 2018:bay119. 10.1093/database/bay119 30576484 PMC6310513

[41] Tantia MS Mishra B Banerjee P Joshi J Upasna S Vijh RK Phylogenetic and sequence analysis of toll like receptor genes (TLR-2 and TLR-4) in buffaloes. Indian J Anim Sci. (2012) 82:875–8.

[42] Gulhane AB Sangwan ML Polymorphism in TLR4 gene and its association with mastitis in Murrah buffaloes. Indian J Biotechnol. (2012) 11:330–2.

[43] Wang X ping Luoreng Z ma Xu S zhong Gao X Li J ya Ren H yan The structure and sequence analysis of TLR4 gene in cattle. Agric Sci China. (2009) 8:632–7. 10.1016/S1671-2927(08)60256-4

[44] Bhattacharya R Rose PW Burley SK Prlić A. Impact of genetic variation on three dimensional structure and function of proteins. PLoS ONE. (2017) 12:e0171355. 10.1371/journal.pone.0171355 28296894 PMC5351996

[45] Zhang H Yin L Wang M Yuan X Liu X. Factors affecting the accuracy of genomic selection for agricultural economic traits in maize, cattle, and pig populations. Front Genet. (2019) 10:189. 10.3389/fgene.2019.00189 30923535 PMC6426750

[46] de Camargo GMF Aspilcueta-Borquis RR Fortes MRS Porto-Neto R Cardoso DF Santos DJA. Prospecting major genes in dairy buffaloes. BMC Genomics. (2015) 16:872. 10.1186/s12864-015-1986-2 26510479 PMC4625573

[47] Cardoso DF de Souza GFP Aspilcueta-Borquis RR Araujo Neto FR de Camargo GMF Hurtado-Lugo NA. Short communication: variable number of tandem repeat polymorphisms in DGAT1 gene of buffaloes (Bubalus bubalis) is associated with milk constituents. J Dairy Sci. (2015) 98:3492–5. 10.3168/jds.2014-8729 25726116

[48] de Freitas AC de Camargo GMF Stafuzza NB Aspilcueta-Borquis RR Venturini GC Dias MM. Genetic association between SNPs in the DGAT1 gene and milk production traits in Murrah buffaloes. Trop Anim Health Prod. (2016) 48:1421–6. 10.1007/s11250-016-1110-x 27469895

[49] Zetouni L de Camargo GMF da Silva Fonseca PD Cardoso DF Gil FMM Hurtado-Lugo NA. Polymorphisms in the MTRN1A gene and their effects on the productive and reproductive traits in buffaloes. Trop Anim Health Prod. (2014) 46:337–40. 10.1007/s11250-013-0493-1 24136157

[50] El Nahas SM Mossallem AAA Abdelhamid MI Warda M. A study on IL8RB gene polymorphism as a potential immuno-compromised adherent in exaggeration of parenteral and mammo-crine oxidative stress during mastitis in buffalo. J Adv Res. (2017) 8:617–25. 10.1016/j.jare.2017.07.002 28819569 PMC5548339

[51] Alfano F Peletto S Lucibelli MG Borriello G Urciuolo G Maniaci MG. Identification of single nucleotide polymorphisms in Toll-like receptor candidate genes associated with tuberculosis infection in water buffalo (Bubalus bubalis). BMC Genet. (2014) 15:139. 10.1186/s12863-014-0139-y 25496717 PMC4278265

[52] Beecher C Daly M Childs S Berry DP Magee DA McCarthy T V. Polymorphisms in bovine immune genes and their associations with somatic cell count and milk production in dairy cattle. BMC Genet. (2010) 11:99. 10.1186/1471-2156-11-99 21054834 PMC3087511

[53] Marchitelli C Savarese MC Crisà A Nardone A Marsan PA Valentini A. Double muscling in marchigiana beef breed is caused by a stop codon in the third exon of myostatin gene. Mamm Genome. (2003) 14:392–5. 10.1007/s00335-002-2176-5 12879361

