# Supplementary material for: Polymorphisms in TLR4 Gene Associated With Somatic Cell Score in Water Buffaloes (Bubalus bubalis)
Source: Front Vet Sci. 2020 Nov 5;7:568249. doi: 10.3389/fvets.2020.568249 (PMC7676892; doi:10.3389/fvets.2020.568249)
Supplement: Supplementary file 1 [file Table_1.DOCX]

**Table S1.** Base divergences and amino acid change in the amplified specific regions of the *TLR4* gene in buffaloes compared to the corresponding sequence in bovine.

| **Locus** | **Pb^#^** | **Divergence** | | **Amino acid change** |
| --- | --- | --- | --- | --- |
| **Region** |  | **Bovine** | **Buffalo** |  |
| Exon 1-5´UTR | 54713 | T | A | - |
| Exon 1-5´UTR | 54697 | G | T | - |
| Exon 1-5´UTR | 54668 | G | T | - |
| Exon 1-5´UTR | 54642 | C | T | - |
| Exon 1-5´UTR | 54573 | C | T | - |
| Exon 1 - CDS | 54398 | T | G | Ser / Ser |
| Intron 1 | 54372 | G | A | - |
| Intron 1 | 49988 | C | A | - |
| Intron 1 | 49936 | C | A | - |
| Intron 1 | 49895 | T | G | - |
| Intron 1 | 49890 | T | C | - |
| Intron 1 | 49857 | C | A | - |
| Intron 1 | 49856 | A | C | - |
| Exon 2 - CDS | 49809 | G | T | Gln / Met |
| Exon 2 – CDS | 49779 | C | A | Pro / Stp |
| Intron 2 | 49633 | A | T | - |
| Intron 2 | 49616 | T | A | - |
| Intron 2 | 49615 | C | A | - |
| Exon 3 - CDS | 46828 | A | G | Gly / Pro |
| Exon 3 - CDS | 46813 | A | G | Leu / Leu |
| Exon 3 - CDS | 46802 | A | C | Gln / Arg |
| Exon 3 - CDS | 46771 | C | T | Val / Val |
| Exon 3 - CDS | 46762 | T | G | Asn / His |
| Exon 3 - CDS | 46739 | A | C | Lys / Arg |
| Exon 3 - CDS | 46737 | A | C | Asn / Ala |
| Exon 3 - CDS | 46736 | A | G | Asn / Ala |
| Exon 3 - CDS | 46614 | T | A | Tyr / Tyr |
| Exon 3 - CDS | 46469 | C | A | Ser / Leu |
| Exon 3 - CDS | 46424 | C | A | Thr / Ile |
| Exon 3 - CDS | 46380 | C | T | Gln / Lys |
| Exon 3 - CDS | 46324 | C | A | Phe / Phe |
| Exon 3 - CDS | 46305 | A | C | Lys / Glu |
| Exon 3 - CDS | 46176 | A | C | Ile / Val |
| Exon 3 - CDS | 46163 | T | A | Phe / Phe |
| Exon 3 - CDS | 46159 | C | T | Asp / Glu |
| Exon 3 - CDS | 45982 | G | T | Lys / Lys |
| Exon 3 - CDS | 45933 | A | C | Met / Val |
| Exon 3 - CDS | 45907 | G | T | Leu / Leu |
| Exon 3 - CDS | 45751 | A | G | Ala / Ala |
| Exon 3 - CDS | 45706 | A | C | Glu / Glu |
| Exon 3 - CDS | 45697 | C | A | Asn / Asn |
| Exon 3 - CDS | 45654 | G | A | Ala / Ser |
| Exon 3 - CDS | 45585 | C | A | Leu / Leu |
| Exon 3 - CDS | 45532 | C | A | Ser / Ser |
| Exon 3 - CDS | 45479 | G | T | Ser / Asn |
| Exon 3 - CDS | 45342 | G | T | Glu / Lys |
| Exon 3 - CDS | 45313 | C | A | Asn / Asn |
| Exon 3 - CDS | 45300 | C | T | Leu / Met |
| Exon 3 - CDS | 45290 | C | A | Thr / Met |
| Exon 3 - CDS | 45277 | G | T | Ser / Ser |
| Exon 3 - CDS | 45274 | G | A | Val / Val |
| Exon 3 - CDS | 45271 | G | T | Val / Val |
| Exon 3 - CDS | 45262 | G | T | Val / val |
| Exon 3 - CDS | 45253 | G | T | Gly / Gly |
| Exon 3 - CDS | 45241 | C | A | Tyr / Tyr |
| Exon 3 - CDS | 45229 | C | A | Gly / Gly |
| Exon 3 - CDS | 45175 | C | T | Val / Val |
| Exon 3 - CDS | 45151 | G | T | Ser / Ser |
| Exon 3 - CDS | 45142 | T | G | Leu / Leu |
| Exon 3 - CDS | 45064 | C | A | Leu / Leu |

* Position of divergences based on the sequence>NW_005784801.1
